# Using Information Theory to Detect Rogue Taxa and Improve Consensus Trees

**DOI:** 10.1093/sysbio/syab099

**Published:** 2021-12-24

**Authors:** Martin R Smith

**Affiliations:** Department of Earth Sciences, Durham University, Durham, UK

## Abstract

“Rogue” taxa of uncertain affinity can confound attempts to summarize the results of phylogenetic analyses. Rogues reduce resolution and support values in consensus trees, potentially obscuring strong evidence for relationships between other taxa. Information theory provides a principled means of assessing the congruence between a set of trees and their consensus, allowing rogue taxa to be identified more effectively than when using *ad hoc* measures of tree quality. A basic implementation of this approach in R recovers reduced consensus trees that are better resolved, more accurate, and more informative than those generated by existing methods. [Consensus trees; information theory; phylogenetic software; Rogue taxa.]

A principal objective of systematic biology is the reconstruction of relationships between evolving lineages, typically depicted as trees. Phylogenetic analyses often return many thousands of individual tree topologies, each of which may be associated with an implicit probability or likelihood. In order to summarize the relationship information represented by such forests, researchers typically present a single consensus tree that depicts groupings of taxa that occur in all or a majority of underlying trees (Adams 1972; Holder et al. 2008).

Ideally, such a consensus tree should be constructed so as to maximize the amount of information about the underlying tree set that is conveyed (Thorley et al. 1998). The information content of a consensus tree—both in terms of its resolution and the support values for individual edges—is greatly reduced by the existence of “rogue” taxa (Wilkinson 1994, 1996, 2003; Wilkinson et al. 1996), whose position varies from tree to tree due to conflict or ambiguity in their associated data (Kearney 2002). In the worst case, the strict consensus of a set of trees that differ only in the position of a single rogue taxon can be a single polytomy that contains no information, whereas a consensus excluding the rogue would perfectly convey the relationships between all other taxa implied by every member of the tree set.

Any taxon or set of taxa whose removal from a tree set increases the information content of a consensus tree can be considered rogue. Aberer et al. (2013) define the “relative bipartition information content” (RBIC) of a consensus tree as the sum of the support values for each of its constituent splits, where a split’s support is defined as the proportion of underlying trees that contain it. Successively removing rogue taxa can result in substantial improvements to the RBIC score (Aberer et al. 2013)—but this scoring method exhibits certain shortcomings that hinder the identification of a truly “optimal” consensus tree (Wilkinson and Crotti 2017). The RBIC approach uses the language, but not the mathematics, of information theory. Using a probabilistic definition of information (Shannon 1948) provides a principled measure of the information content of consensus trees (Thorley et al. 1998; Wilkinson et al. 2004a), which I suggest might improve the identification of rogue taxa.

## Methods

### Definitions

A *phylogenetic tree* is an acyclic graph comprising *leaves* (labeled nodes of order one) and *internal nodes*, with an order greater than two. Nodes of order three are *fully resolved*, whereas nodes of higher order are *polytomies*. A tree in which each internal node is fully resolved is *binary*. In a *rooted* tree, one edge is designated as the “root.”

Various indices have been proposed to measure the stability of leaves between trees (e.g., Thorley and Wilkinson 1999; Thomson and Shaffer 2010). I define the *instability* of a pair of leaves as the natural logarithm of the median absolute divergence in their graph geodesic (the number of edges in the shortest path between the leaves) across all trees, normalized against the mean graph geodesic between those leaves across all trees. The instability of a single leaf is the mean instability of all pairs that include that leaf; higher values characterize leaves whose position is more variable between trees. Taking a logarithm emphasizes changes in leaf-to-leaf distance when a leaf is moved a large distance over smaller changes arising due to, for example, changes in tree shape that have limited impact on inferred relationships.

Each edge in a phylogenetic tree represents a unique *split* $S = A| B$ which bipartitions all leaves into two disjoint subsets. The *majority rule consensus* of a set of trees is a single tree that contains only the splits that occur in a majority of trees. The *support* for a split is its frequency in a set of trees, which is taken here as a proxy for the implied *probability* that the split is *true*, that is, that it occurs in the (potentially unknown) true tree, given the data from which the trees were generated. This is the literal interpretation of split support where trees represent a Bayesian posterior sample, but represents an ambitious interpretation of bootstrap support values (Berry and Gascuel 1996), and should be applied only reluctantly to sets of most parsimonious trees.

The Shannon (1948)*information content* of an observation, measured in bits, is $-\log_2 p$, where $p$ is the probability of the observation. The Shannon *entropy* of a set is the mean information content of all elements of the set, $H( X) = \sum-p_i \log_2 p_i$, which simplifies to $\log_2 (| X |)$ for a set $X$ of equiprobable elements (MacKay 2003).

There are $R(n) = 2n-3!!$ rooted trees and $U(n) = 2n-5!!$ unrooted trees on $n$ leaves, where the double factorial $x!! = x\times(x-2)!!$, and $0!! = 1!! = 1$. The split $S = A| B$ occurs in $U_{A\mid B} = R(| A|) + R(| B |)$ unrooted trees, and is absent in the remaining $U_{A\nmid B} = U(| S|)-U_{A\mid B}$ trees.

The *phylogenetic information content* (*sensu*Thorley et al. 1998) of the split $S = A| B$ is the difference in entropy imputed by the split on the set $X$ of all binary $n$-leaf trees, that is, $H( X)-H(X|S)$. Let split $S$ be true with probability $p$ and false with probability $q = 1-p$. The probability of all $U_{A\mid B}$ trees that contain $S$ sums to $p$; taking all such trees to be equiprobable, any individual tree has probability $\frac{p}{U_{A\mid B} }$ and an information content of $- \log_2 \frac{p}{U_{A\mid B} }$. Likewise, the information content of any individual tree that does not contain $S$ is $\log_2 \frac{q}{U_{A\nmid B} }$. The entropy of $X$ given $S = A| B$ is thus

$$
\begin{align*}
H(X| S) =-p\log_2 \frac{p}{U_{A\mid B} }-q\log_2 \frac{q}{U_{A\nmid B}}
\end{align*}
$$

and its phylogenetic information content is

$$
\begin{align*}
H( X)-H(X| S) &= \log_2 U(| S|) + p(\log_2 p-\log_2
U_{A\nmid B})\\
&\quad + q(\log_2 q-\log_2 (U_{A\nmid B}))\mbox{ bits}.\end{align*}
$$

The *splitwise information content* of a tree is the sum of the information content of each split in turn. Because splits are not independent, this quantity counts some information multiple times; it is nonetheless a useful proxy for the total information content of a tree (Smith 2020) and can be calculated in polynomial time. I propose using the *splitwise phylogenetic information content* (SPIC) to score a consensus tree, such that a tree with the highest SPIC score is considered *optimal*. This indirect approach is used in place of the straightforward cladistic information content of a cladogram (Thorley et al. 1998) in order to incorporate the uncertainty associated with split support values under $100{\%}$.

### Finding an Optimal Consensus Tree

I compared two heuristic approaches to finding an optimal consensus tree.

Starting with the input trees, the first heuristic (H1) deletes the leaf with the highest instability value (breaking ties arbitrarily) and generates the consensus of the resulting trees. This step is repeated, without replacing dropped leaves, until dropping further leaves cannot possibly result in a consensus tree that contains more information than the best yet found. Moving to the consensus tree with the highest splitwise phylogenetic information content, the method then considers the most recently deleted leaf. If reinstating that leaf in its original position in each of the underlying trees increases the information content of their consensus, then the leaf is retained; otherwise, it remains deleted. This step is repeated for each other leaf that was removed, working from the most recently deleted to the least. H1 can be implemented in $O(n_{\text{leaves}}^2 + n_{\text{trees}}^2)$ time.

The second heuristic (H2) follows an approach implemented in “RogueNaRok” (Aberer et al. 2013). The SPIC of the majority-rule consensus tree is taken as a starting score. Then, each combination of $1..d$ leaves in turn is selected as a *dropset*, and a consensus tree is constructed that omits all leaves in that dropset. If any such consensus tree contains more information than the best yet found, then the members of whichever dropset yielded the most informative tree are deemed rogues, and removed from each tree in the tree set. Subsequent iterations are performed until no dropset of up to $d$ leaves gives rise to a more informative consensus tree. H2 can be implemented in $O(n_{\text{leaves}} ^{d + 1}\times n_{\text{trees}} ^2)$ time.

Heuristics H1 and H2 are implemented alongside an interface to the “RogueNaRok” library (Aberer et al. 2013) in the R (R Core Team 2021) package “Rogue,” available via the Comprehensive R Archive Network. Selected functions are integrated into the “TreeSearch” graphical user interface (Smith 2021).

### Evaluation

Aberer et al. (2013) compared approaches to rogue identification by simulating 400 data sets of DNA sequences for approximately 100, 200, or 500 taxa, using the generalized time-reversible model (Tavaré 1986) under four different sets of parameters representative of real-world data sets; then for each data set, generating 200 bootstrap trees using RAxML (Stamatakis 2006). The tree from which DNA sequences were simulated was treated as the “true” reference tree.

I analyzed each set of bootstrap trees under each heuristic method in order to identify rogue taxa, using a maximum dropset size of two for heuristic H2. As the 500-leaf tree sets exhibited long run times under heuristic H2, only the 100-leaf and 200-leaf tree sets were analyzed with this approach. A consensus tree was constructed after dropping the $n$ rogues identified by each analysis. For context, a second consensus tree was generated after dropping $n$ randomly selected leaves. Results were compared with previously reported results for “RogueNaRok” using a maximum dropset size of three (Aberer et al. 2013).

The principal criterion against which a consensus tree should be evaluated is how well it reflects the information in the trees it summarizes—whose similarity to a “true” tree will depend on the properties of the method and data used to generate the tree set. To evaluate consensus trees, for each tree in the tree set, I removed any leaf not present in the consensus tree, then subtracted the information content of any split explicitly contradicted by the consensus tree from the sum of the information content of each split held in common with the consensus tree. The resulting total was normalized against the total splitwise information content of all splits in the original tree set. Separately, I calculated the net quartet similarity—that is, the number of quartets resolved the same way minus the number resolved differently—between the consensus tree and each member of the original tree set.

Aberer et al. (2013) evaluated consensus trees based on their similarity to the known tree topology used to generate data sets, assuming the underlying tree set to adequately reflect the “true” tree topology. To usefully inform phylogenetic conclusions, a summary tree should be both precise (i.e., resolve many informative splits) and accurate (i.e., resolve splits correctly) (e.g., Smith 2019a). To measure precision, I calculated the splitwise phylogenetic information content of each consensus tree ($Inf_{\rm total}$). To measure the phylogenetic *signal* contained within a tree, I calculated the amount of phylogenetic information shared between the splits in the consensus tree and the “true” tree when splits are optimally matched ($Inf_{\rm signal}$, the shared phylogenetic information score of Smith (2020)). I classified the information that is not signal ($Inf_{\rm total}- Inf_{\rm signal}$) as *noise*. Subtracting the amount of noise from the signal gives a measure of accuracy that accounts for differences in resolution: an unresolved tree will have zero accuracy; resolving more relationships correctly increases accuracy, whereas resolving additional relationships incorrectly reduces accuracy: it is preferable to have no information than to have misleading information (Holder et al. 2008).

In order to pinpoint the effect of removing rogue taxa on tree quality, all results are presented as differences from the scores of the plenary consensus tree (Wilkinson et al. 2004b)—that is, the consensus of all trees before the removal of rogues.

As a complementary measure of tree similarity, I used “tqDist” (Sand et al. 2014) and “Quartet” (Smith 2019b) to count the number of quartets (Estabrook et al. 1985) in the consensus tree that were unresolved ($u$), resolved in the same way ($s$), or resolved differently ($d$), to each corresponding quartet in the true tree, and to each tree in the underlying tree set. As tqDist only supports trees with $<$478 leaves, trees in the 500-leaf set could not be analyzed by this method.

For simplicity, the evaluation of precision and accuracy considered only the topology of each majority rule consensus tree, without incorporating measures of split support—though doing so did not materially affect the outcome. The same pattern of results was also evident when using clustering information (Smith 2020) rather than phylogenetic information to evaluate tree similarity and information content.

## Results

**Figure 1.**
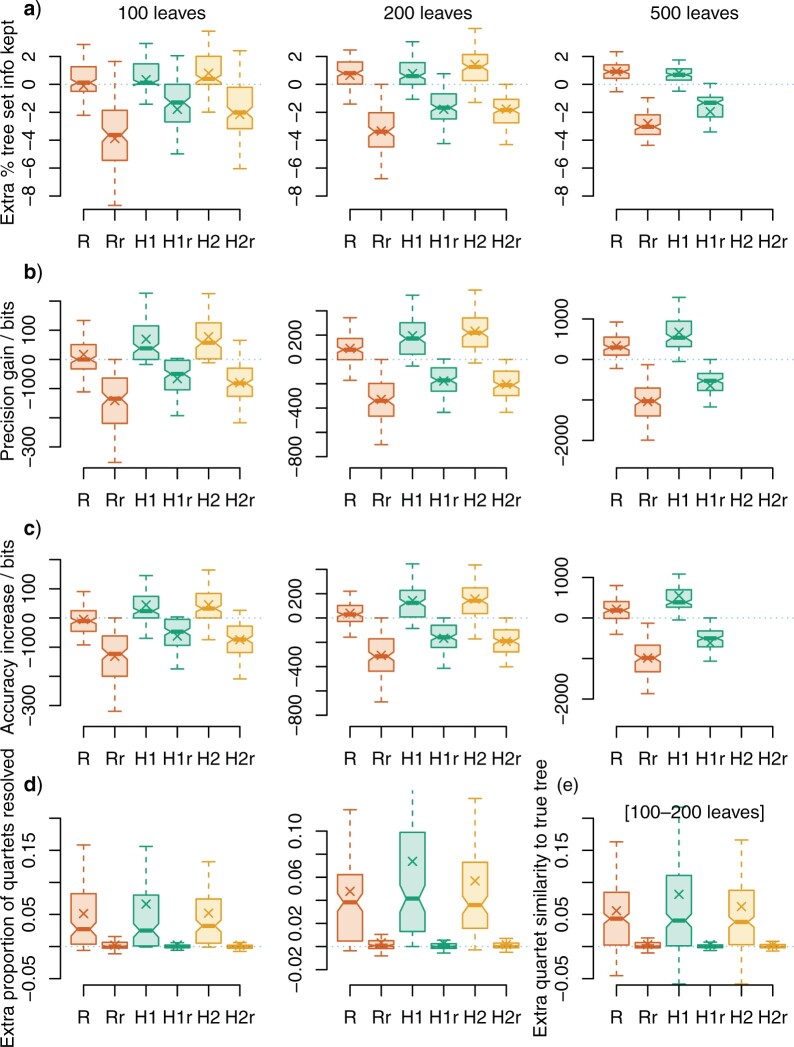
Performance of rogue-detection approaches. Changes in consensus tree quality after removing rogue taxa. Rogues were identified using relative bipartition information content (“R,” using RogueNaRok) and shared phylogenetic information content (heuristics H1, H2); or by selecting the same number of leaves at random (-$r$ suffix). Zero indicates no change in quality from the plenary consensus. a) extra percentage of information retained from tree set: phylogenetic information content (PIC) of retained splits, minus PIC of contradicted splits, normalized against total PIC of all splits; b) extra precision (total PIC of all splits in consensus); c), extra accuracy (PIC of “true” splits minus PIC of “false” splits); d) extra proportion of quartets that are resolved (i.e., precision); e) extra accuracy (“true” quartet statements minus “false” quartet statements), trees with c. 100–200 leaves. Columns in a–d correspond to data sets with c. 100, c. 200, and c. 500 leaves. Crosses denote means; hinges denote interquartile range; whiskers extend to the most extreme data point that is no more than 1 interquartile range from the box. Differences between medians (bars) are significantly different at the $5{\%}$ level where notches do not overlap (Chambers et al. 1983).

All methods of rogue detection increased the mean amount of information from the underlying tree set reflected in the consensus tree, with the SPIC heuristic H2 producing consensus trees that are most representative of the underlying trees (Fig. 1a). Under each method, deleting an equivalent number of leaves at random resulted in a consensus tree that was less concordant with the underlying tree set (-$r$ suffix in Fig. 1).

The SPIC heuristics consistently improved the precision of consensus trees (Fig. 1b) and their congruence with “true” reference trees (Fig. 1c). Despite its larger maximum dropset size, “RogueNaRok” produced much smaller gains; indeed, with 100-leaf trees, it produced no median improvement in precision and a decrease in mean and median accuracy. Gains in resolution obtained by omitting rogue taxa identified by “RogueNaRok” were largely offset by the loss of all information regarding the position of the rogues.

Under quartet measures, the relative resolution of trees—the proportion of quartets in a tree that are resolved—is improved by deleting rogues, but not by deleting leaves at random. The three rogue detection methods produce the same median improvement (Fig. 1d), but the mean improvement under the SPIC criterion is higher than that accomplished using the RBIC; heuristic H1 produces the highest mean resolution. Relative accuracy (the proportion of quartets resolved “correctly” minus the proportion resolved “incorrectly”) displays the same pattern, largely because the proportion of quartets resolved “incorrectly,” relative to either the true tree or trees in the underpinning tree set, is small: as the proportion of incorrect quartets tends to zero, the two measures converge. Low numbers of “incorrect” quartets reflect the lower sensitivity of the quartet approach to individually misplaced leaves relative to bipartition-based measures.

Overall, the fast H1 heuristic identifies rogue taxa as well as or better than the “RogueNaRok” heuristic with a dropset size of three, with the additional benefit of a shorter run time (Table 1). Rogue detection is better still under the H2 heuristic, at the cost of substantially higher time and memory requirements as presently implemented.

**Table 1.** Run time and memory requirements when detecting rogue taxa in three data sets with the “Rogue” R package, using: R, RBIC, via RogueNaRok; H1, SPIC heuristic 1; H2, SPIC heuristic 2

	Leaves	Dropset size	Time (s)	Cumulative memory use (Mb)
R	99	1	47.3	53
R	99	2	50.9	49
R	99	3	57.5	50
H1	99	—	2.1	816
H2	99	1	4.9	1541
H2	99	2	84.7	30,518
H2	99	3	2252.6	826,750
R	200	1	1.0	103
R	200	2	12.3	97
R	200	3	142.0	96
H1	200	—	11.5	4214
H2	200	1	77.1	25,081
H2	200	2	686.0	230,240
H2	200	3	20,626.8	7,166,800
R	490	1	10.1	225
R	490	2	398.9	222
R	490	3	17,457.6	223
H1	490	—	160.1	94,212

## Discussion

Alongside its better performance on empirical data sets, the SPIC score exhibits additional properties that make it a suitable tool for evaluating consensus trees.

**Figure 2.**
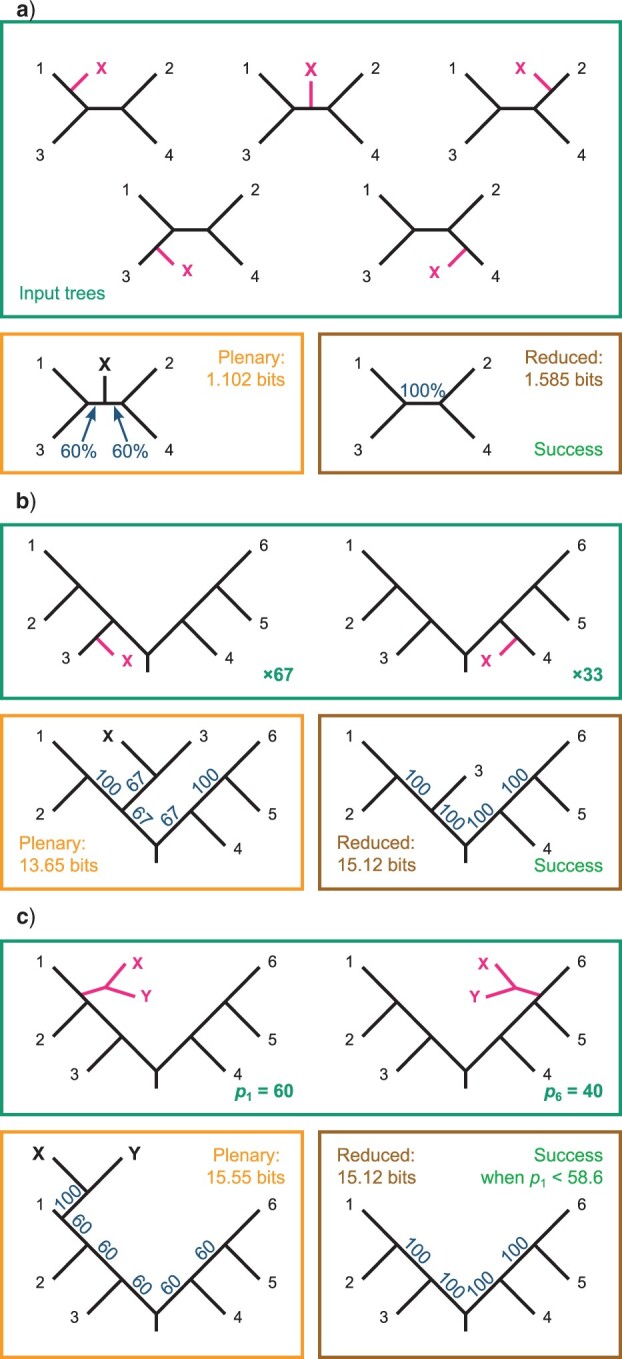
Evaluation of tree pairs presented by Wilkinson and Crotti (2017) using the SPIC score. a) A single rogue leaf X may occupy any of the five possible positions on a four-leaf tree; the reduced consensus reveals the unanimous agreement about the relationships between taxa 1–4 and is thus preferred to the plenary. b) Rogue leaf X is sister to leaf 3 in 67 trees and leaf 4 in 33 trees; the reduced consensus reveals unanimous agreement about relationships between taxa 1–6 and is preferred to the plenary consensus. c) A rogue clade comprising two leaves X and Y is sister to leaf 1 with probability $p_{1} = 60{\%}$ and sister to leaf 6 with probability $40{\%}$. The plenary retains the information that X and Y form a clade at the cost of lower branch supports elsewhere in the tree; the reduced consensus reveals unanimity in the relationships between leaves 1–6 but contains no information about leaves X and Y. The SPIC score prefers the reduced consensus only when the position of X and Y is uncertain ($p_{1}< 58.6{\%}$).

Wilkinson and Crotti (2017) demonstrate examples where the RBIC measure employed by RogueNaRok (Aberer et al. 2013) undesirably favors one topology over another; the SPIC score ranks these tree pairs correctly (Fig. 2).

A further advantage of the SPIC over the RBIC criterion is that removing a leaf is associated with an intrinsic cost. If a tree contains a polytomy, then removing one leaf from that polytomy will only impact the RBIC score if it results in changes to node supports elsewhere in the tree, even though the removal means that the tree no longer contains any information about the possible relationships of that leaf—information that is an intrinsic component of a tree’s splitwise phylogenetic information content score.

This consideration serves to militate against the removal of large rogue clades, as doing so removes not just any relationship information within the clade, but also the information that the affected taxa are monophyletic. By way of example, Wilkinson and Crotti (2017) envisage a situation in which a rogue pair of taxa occur at one of two opposite points on a balanced rooted six-leaf tree (Fig. 2c). Under my information-theoretic framework, the information that the pair of taxa are each other’s closest relatives can outweigh the information lost when the leaves are included in a consensus tree (reducing the support values of other clades), but only where one of the two positions occurs with a frequency greater than $58.5{\%}$.

Perhaps because of its grounding in information theory, the SPIC score can be used to obtain consensus trees that are more informative than those produced by optimizing the RBIC. Finding the reduced consensus tree with the highest SPIC score is nevertheless a nontrivial problem. Whereas the dropset-based approach of H2 often finds a better set of rogues than H1, its present implementation is resource intensive. Much of the difference in run time between RogueNaRok and the SPIC implementation in “Rogue” can be attributed to aggressive optimization of the RogueNaRok subroutines (Aberer et al. 2013), and their implementation in exclusively in C, rather than a combination of R and C++. Similar optimization might be expected to reduce the computational cost of the H2 approach substantially. Alternatively, the relatively strong performance of H1 demonstrates the promise of computationally simpler heuristic approaches. Further improvements may be possible by modifying aspects of this approach, for example by using other quantifications of leaf stability (e.g., Estabrook 1992; Thorley and Wilkinson 1999; Pol and Escapa 2009; Goloboff and Szumik 2015), or by integrating other heuristic methods, such as a “genetic” evolutionary approach (Srivastava et al. 2018).

## Conclusion

I have shown that an information-theoretic definition of information can provide an improved means of evaluating consensus trees, and thus the detection of rogue taxa—despite the repeated counting of information that results from treating splits as independent, which might in future be circumvented through the application of hierarchical measures of information (Perotti et al. 2015, 2020).

Whenever a consensus tree exhibits low resolution or low branch support values, I recommend searching for rogue leaves using a method that aims to maximize the SPIC score. The existence of rogues militates for a detailed evaluation of optimal trees. The only way to fully evaluate the signal within a set of trees is to scrutinize each individual topology, perhaps assisted by an exploration of the underlying tree space (St. John 2017; Smith 2022) and the construction of a “profile” of complementary consensus trees (Wilkinson 1996; Wilkinson 2003).

Nevertheless, removing rogues thus identified—perhaps in combination with *a priori* identification of noninformative taxa (Wilkinson 1995) and depiction of the position of omitted rogues (e.g., Smith and Dunn 2008; Klopfstein and Spasojevic 2019)—allows the construction of a consensus tree that optimally summarizes the phylogenetic signal in a set of rogue-bearing phylogenetic trees.
